# Oral Syphilis: A Reemerging Infection Prompting Clinicians' Alertness

**DOI:** 10.1155/2016/6295920

**Published:** 2016-05-18

**Authors:** Sebastian Dybeck Udd, Bodil Lund

**Affiliations:** ^1^Department of Oral and Maxillofacial Surgery, Karolinska University Hospital, 171 76 Stockholm, Sweden; ^2^Department of Dental Medicine, Karolinska Institutet, Box 4064, 141 04 Huddinge, Sweden

## Abstract

Syphilis is a rare but increasing disease. Due to changing sexual habits, presentation of oral manifestations may rise. Since syphilis may mimic other oral manifestations, diagnoses can be difficult. Clinicians need to be aware that ambiguous oral manifestations may in fact be caused by oral syphilis. Here, we present a case of extended diagnostic delay highlighting the importance of consulting an expert in infectious diseases in case of obscure oral lesions not responding to standard treatment. Despite seven visits to six different medical doctors, a patient who presented with oral syphilis was continuously misdiagnosed. After 6 months of increasing complaints and deteriorating severity of disease, the patient was referred to an oral and maxillofacial surgeon where the correct diagnosis was determined and proper treatment initiated.

## 1. Introduction

Syphilis is a rare disease that after a global decrease over the past several decades now displays reemergence [1]. Syphilis is caused by a spirochete bacteria,* Treponema pallidum* subsp.* pallidum*, and is according to Swedish regulations a reportable disease. Syphilis presents in three stages denoted as primary, secondary, and tertiary syphilis. The primary lesion appears at the site of infection and is characterized by healing ulcers. Varying periods of latency may occur between the stages with the risk of rendering the treating physician with the erroneous illusion of successful treatment in case of misdiagnosis [2]. Since the secondary stage is due to systemic spread of the spirochetes beyond the primary infection site, early treatment during primary stage is important. Oral presentation of syphilis, such as ulceration, mucous patches, and maculopapular lesions, is most commonly occurring at the secondary stage and is more seldom a sign of primary disease [1–3]. Changes in sexual habits such as increasing practice of fellatio can make the primary chancre appear in the mouth. Approximately one-third of the patients proceed into a tertiary stage. Involvement of the central nervous system like cognitive symptoms, ataxia, and paralysis may occur in all stages but is often associated with the tertiary stage. Further typical manifestations of the tertiary stage are gumma and generalized glossitis. A cardiovascular syphilis may also occur including aortitis and coronary ostial stenosis with the risk of aneurysm and angina pectoris, respectively.

Since* T. pallidum* is not cultivable, serology, in combination with a thorough clinical examination, is commonly used for diagnosis [4]. Empiric treatment prior to diagnosis hampers the tracing of infectious diseases in the society promoting intraindividual dissemination. If not diagnosed under the first or second stage, the patient may be subjected to long-term carriage with the risk of fatal complications. These rare manifestations, that may mimic other diseases, demand skilled and alert clinicians for proper and prompt diagnosis.

This case report highlights the importance of consulting a doctor well trained in infectious disease for patients with ambiguous oral manifestations to avoid a delay in treatment with the consequences of unnecessary suffering and risk of permanent damage and disease transmission.

## 2. Case Report

A 53-year-old man seeks a general practitioner at a primary health care clinic with main complaints of sore throat and burning sensation in the pharyngeal area. A fungal infection was suspected and antifungal medication was prescribed. Two weeks later, the patient reappears at the clinic with no improvement. The patient was referred to an ear, neck, and throat (ENT) specialist. By this time the patient had, besides the oropharyngeal complaints, developed genital rash. Again fungal infection was suspected but not verified by culture. Besides antifungal treatment, the patient was also given erythromycin for unclear reason. It was appraised that the condition did not require any follow-up. Two months later, the patient visits a hospital emergency clinic with remaining ulcers and erythematous lesions of the oral cavity and genital area and emerging rash and red macules on hands, foot soles, and abdominal region. The patient was tested negative for HIV and* Chlamydia* spp. infection and again a fungal infection was suspected. A third antifungal treatment was prescribed, this time with the addition of topical cortisone. The patient was being told that the condition most likely was stress related. Approximately one month later, the patient visits an emergency ENT-clinic showing deteriorating oral manifestations in the form of painful ulcers which then were considered as aphthous-stomatitis. Again the condition was regarded as stress related and the patient was empirically prescribed per oral phenoxymethylpenicillin. Despite request by the patient, he was denied a referral to a clinic for sexual transmitting diseases (STD), since the diagnosis of aphthous-stomatitis was considered verified. On his own initiative, the patient sought care at a STD-clinic but was denied appointment with the motivation that a written referral was required. Another month later, the patient visits his local health care physician suffering from previously described symptoms with the addition of dizziness/vertigo. Again the condition was considered to be stress related. Yet another month later, the patient once again visits his general practitioner describing symptoms including difficulties in memory and perceived affected cognition. For the fourth time, the symptoms were considered to be stress induced.

**Figure 1:**
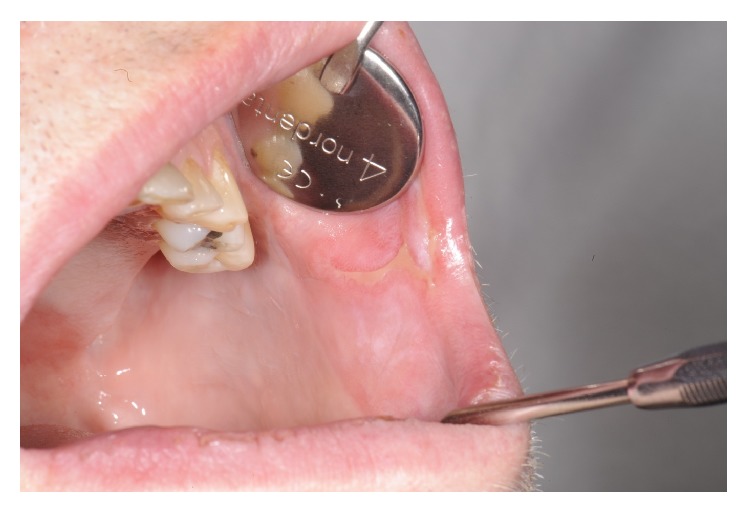
Clinical appearance of buccal ulceration.

**Figure 2:**
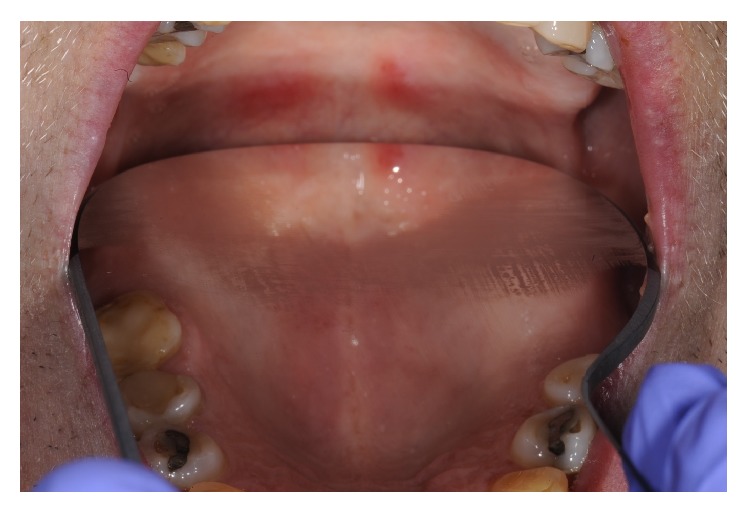
Clinical appearance of erythematous lesions in the soft palate.

Altogether more than six months after the debut of symptoms the patient visits the Institute of Odontology at Karolinska Institutet for a regular annual dental examination. Because of the oral manifestations, an appointment at the Oral and Maxillofacial Surgery Department was urgently arranged. The oral examination showed erythematous lesions of the soft palate and also ulceration of the left buccal mucosa (Figures 1 and 2). At this point, the patient stated that the lesions a few weeks earlier had been more severe in size and symptoms engaging the entire oral cavity. After a thorough patient history, including general health and sexual habits, combined with clinical investigation, the tentative diagnosis oral syphilis was suspected. The patient was referred to a STD-clinic where diagnosis was confirmed via serology. The sample was screened with the specific tests chemiluminescent microplate immunoassay (CMIA) and confirmed positive with* T. pallidum* particle agglutination assay (TPPA). The patient was treated with intramuscular injections of benzyl-penicillin and antibody titers were monitored with the unspecific test Venereal Disease Reference Laboratory (VDRL). Shortly, after the patient experienced rapid improvement regarding all symptoms, within some weeks, the patient was totally devoid of disease manifestations and declared free from syphilis. The index patient was identified and also subjected to treatment according to Swedish infectious disease regulations.

## 3. Discussion

This case report highlights the reemergence of syphilis and emphasizes the importance of considering syphilis as a case of oral manifestation of unclear origin. The described six-month diagnostic delay, despite several contacts with different medical doctors, leads to a disease progress to secondary stage of syphilis. It was not possible to set the exact time for primary infection due to the patient's sexual behavior characterized by multiple partners including men. If syphilis is not diagnosed during the second stage, it is likely to remain undetected for a substantial time period which may have fatal consequences. Importantly, untreated patients in primary or secondary stage of syphilis are to be considered as contagious [1, 3].

The diagnostic flaws in the described case included suspected fungal infection. This should have been verified by culture, if not initially, then definitely after failed treatment. Moreover, before considering psychological, or stress related, causes of a certain condition all other possible physiological explanations should be ruled out. Antibiotics were prescribed twice without clear indications. Although such treatment may by chance be correct, the patient is likely to be reinfected because lack of diagnosis prevents the index patient from being detected. Thus, in the current case, repeated reinfection cannot be ruled out, which emphasizes the importance of diagnoses prior to antibacterial treatments. Another risk when dealing with oral lesions caused by syphilis is relying on biopsy based diagnostics, since routine histologic appearance of syphilis infected tissue is mainly an unspecific inflammatory reaction. The first choice for such conditions is often corticosteroid which might trigger acute exacerbation of the infection. The extensive periods of latency that may occur can give the false impression of successful treatment.

Though still infrequent, the significant increase in occurrence in combination with altered sexual behavior in the modern society warrants future alertness in case of unclear oral lesions. Because of today's successful treatments for HIV rendering the patient more or less noncontagious, practice of unsafe sex might increase.

In conclusion, in patients with ambiguous oral manifestations, oral syphilis should be ruled out and preferentially the patient should be referred to a physician well trained in infectious diseases.
